# Subcutaneous Sweet Syndrome Presenting as Cellulitis in a Pregnant Female

**DOI:** 10.7759/cureus.17999

**Published:** 2021-09-15

**Authors:** Matt Turner, Krati Chauhan

**Affiliations:** 1 Dermatology, Southern Illinois University School of Medicine, Springfield, USA; 2 Internal Medicine, Rheumatology, Southern Illinois University School of Medicine, Springfield, USA

**Keywords:** vasculitis, subcutaneous tissue, cellulitis, pregnancy, sweet syndrome

## Abstract

We report a case of subcutaneous Sweet syndrome in a pregnant woman that was initially believed to be cellulitis. She was admitted after failure of symptom resolution following multiple oral antibiotics as an outpatient. Her rash continued to progress, and she became nauseous with a lack of appetite. Infectious disease, rheumatology, and dermatology were consulted. Skin biopsies were taken, and while awaiting results, due to continued disease progression despite broad-spectrum antibiotic coverage, IV steroids were started with rapid resolution. Eventual biopsy results showed a dense neutrophilic infiltrate in the subcutaneous fat in a lobular distribution without evidence of vasculitis, confirming a diagnosis of subcutaneous Sweet syndrome. This disease is exceedingly rare in pregnant patients, with few reported cases.

## Introduction

Subcutaneous Sweet syndrome (SSS) is a variant of classic Sweet syndrome, a rare febrile neutrophilic dermatosis. Patients usually present with erythematous nodules and plaques, commonly at the extremities. These lesions may ulcerate and drain oily fluid. In addition, patients may have accompanying fever, malaise, and joint pains. The etiology of this disease is unknown; however, it has been associated with inflammatory conditions, certain drugs, neoplastic disease, and pregnancy [1]. Pregnancy-associated Sweet syndrome is known to make up only 2% of Sweet syndrome cases and to our knowledge, only few such cases have been reported in the literature [2-4]. Thus, we present a case of the subcutaneous variant occurring during pregnancy that originally manifested with characteristics more typical of cellulitis.

## Case presentation

**Figure 1:**
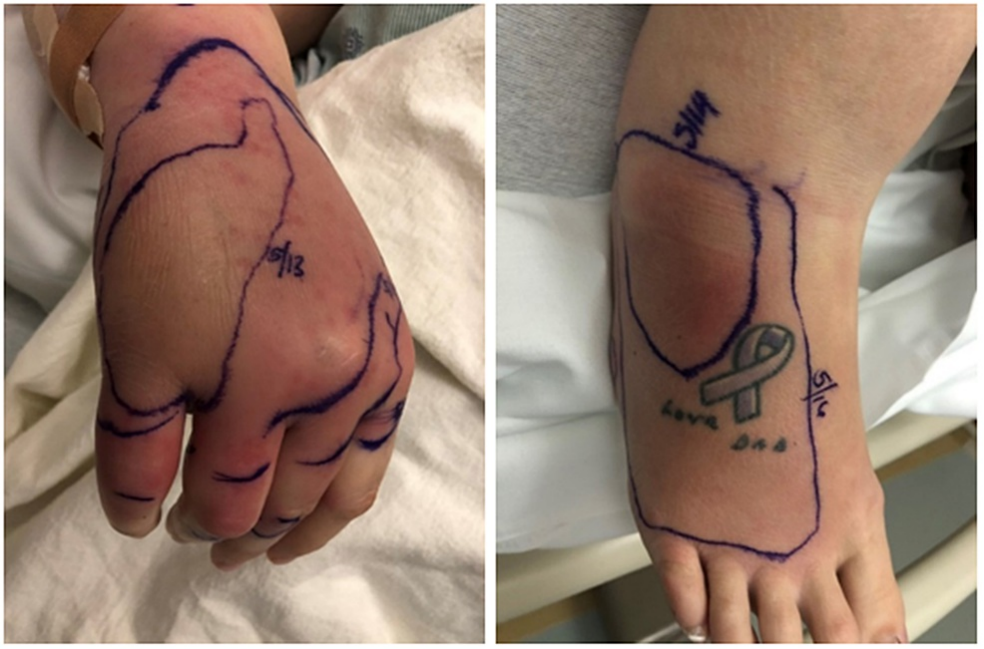
Erythematous and edematous rash on right hand and right ankle

A 35-year-old G7P2 female at 12 weeks gestational age presented to the emergency department with a six-day history of worsening rash and swelling of her right hand and ankle (Figure 1). This started at the right fifth finger and progressed to involve the dorsum of the same hand, along with the second to fifth metacarpophalangeal (MCP) joints. There was associated pain throughout and decreased range of motion of the right wrist and hand joints. The patient noted recent chills and body aches but no fever. She had originally seen her family physician two days prior and was prescribed cephalexin and ceftriaxone for a presumptive diagnosis of cellulitis. On a subsequent emergency room visit the next day for no change in the disease course she was prescribed clindamycin, once again with no improvement.

On presentation to the inpatient unit, due to infectious disease, her antibiotics changed to daptomycin and meropenem for broad coverage. Further history included no recent travel, infection, or exposure to pets. She denied insect bites and close contacts with similar symptoms. Her medical history was significant for hypertension, treated with lifestyle changes, and gestational diabetes. Social history was negative for smoking and drug use, and she had no family history of autoimmune disease. There was no history of sexually transmitted infections. Physical examination revealed an edematous, light pink plaque on the right dorsal hand and wrist that spread proximally up her arm with satellite firm, pink papules on the forearm. Numerous similar appearing papules were present on the left forearm. A small lesion was present near her right fifth MCP that had a gray, dusky appearing hue. On her right foot, there was a dark red/tan edematous plaque. Lab results on admission included an unremarkable complete blood count (CBC) and comprehensive metabolic panel (CMP). Differentials considered were broad, and included cellulitis, parvovirus, Lyme disease, disseminated gonococcal infection, and autoimmune etiologies.

**Figure 2:**
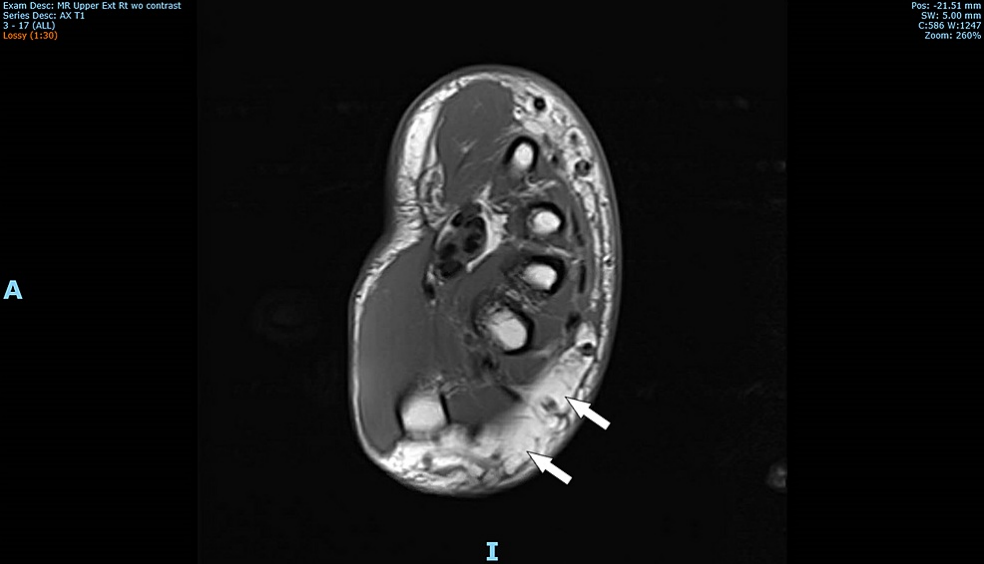
Axial MRI of right upper extremity revealing marked subcutaneous edema

Over the course of her hospital stay, her condition continued to worsen. This included the onset of nausea and loss of appetite. Rheumatology and dermatology were consulted. An x-ray of the right hand showed no acute findings and was followed by an MRI that showed marked subcutaneous edema, possible fourth finger flexor tendon tenosynovitis, and no evidence of infection or abscess (Figure 2). Erythrocyte sedimentation rate (ESR) and C-reactive protein (CRP) were elevated; however, autoimmune antibody testing, blood cultures, Lyme serology, gonococcal testing, and parvovirus IgM were all negative. Trans-thoracic echocardiogram showed no evidence of valve vegetations. Punch biopsies of the right wrist and left forearm were obtained on the second day post-admission.

As the patient's rash was progressing despite empiric antibiotic therapy and all cultures were negative, the antibiotics were discontinued on the third day post-admission and empiric IV steroids were begun. IV methylprednisolone 40 mg once daily was started which led to rapid improvement in her rash, swelling, nausea, and loss of appetite within 24 hours.

**Figure 3:**
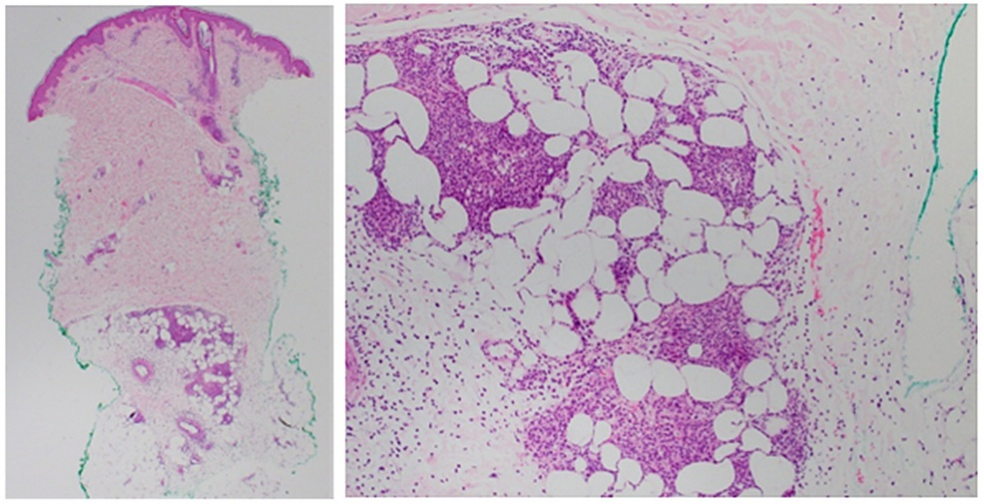
Punch biopsy showing a neutrophilic infiltrate in the subcutaneous fat lobules

Eventual punch biopsy results obtained after discharge showed an inflammatory cell infiltrate consisting of neutrophils in the subcutaneous fat lobules (Figure 3). There was no vasculitis, and special stains and cultures were negative. Following these results, she was diagnosed with subcutaneous Sweet syndrome.

## Discussion

Subcutaneous Sweet syndrome, a variant of classic Sweet syndrome, is a rare neutrophilic dermatosis that presents with lesions that encompass a wide differential diagnosis. In addition to bacterial cellulitis, this list also includes erythema multiforme (EM), erythema nodosum (EN), Behçet syndrome, pyoderma gangrenosum, among others [2].

EM is a hypersensitivity reaction, usually from infection, that presents as erythematous targetoid lesions. When considering EM, oral lesions are more common than in Sweet syndrome. These start as pseudopustular in presentation and evolve to aphthoid lesions [2]. EN lesions appear as painful well-demarcated erythematous red-brown subcutaneous nodules that commonly affect the anterior lower legs and can appear identical to those found in Sweet syndrome. Behçet syndrome is a presumed autoimmune disorder characterized by the presence of genital ulcers, uveitis, and skin lesions such as EN. Pyoderma gangrenosum is another neutrophilic dermatosis that appears as a rapidly enlarging ulcer with a violaceous border. Pathergy may be found in all three of Behçet syndrome, pyoderma gangrenosum, and Sweet syndrome [2]. Subcutaneous sarcoidosis should also be considered which may present as firm flesh-colored or violaceous subcutaneous nodules.

Our patient presented with a rapidly spreading painful, erythematous rash that appeared to be consistent with cellulitis. She had no such symptoms in any of her prior pregnancies. A review of her medications identified no drugs known to be associated with SSS. She was treated with three doses of IV methylprednisolone 40 mg daily and a two-week oral steroid taper with rapid resolution of symptoms.

Pregnancy-associated subcutaneous Sweet syndrome is exceedingly rare. As previously noted, the etiology is unknown, however, in the case of pregnancy, elevated estrogen and progesterone levels are thought to play a role in the immunological changes that result in the disease [5].

It is important to note that in many cases, Sweet syndrome is associated with underlying malignancy. A 1993 review found that approximately 21% of patients diagnosed with Sweet syndrome either had already been diagnosed or were subsequently diagnosed with a malignancy [6]. Most often these malignancies are hematologic in nature, including acute myeloblastic leukemia, myeloproliferative neoplasms, diffuse large B-cell lymphoma, and Hodgkin lymphoma [6,7]. Sweet syndrome is also found with upper respiratory tract and gastrointestinal infections, and inflammatory bowel disease [1]. Other associations exist such as infections like HIV and tuberculosis, rheumatoid arthritis, and sarcoidosis; however, these findings are less frequent and less definitive [1]. Our patient had no history of malignancy.

Histopathologic evidence is needed to make the diagnosis of SSS [2]. As seen in our patient, a dense neutrophilic infiltrate will be seen in the subcutaneous fat. This will primary be in a lobular distribution, although septal involvement may be seen.

## Conclusions

We report a case of subcutaneous Sweet syndrome in a pregnant patient that appeared to be cellulitis at original presentation. Although this disease is rare in the general population, and even more so in pregnant patients, we recommend keeping a wide differential in such patients with a rash, and to have a low threshold for consideration of skin biopsy if symptoms show no improvement with empiric antibiotics given for presumed bacterial cellulitis.
